# Dynamic Shifts of Heavy Metals During Mixed Leaf Litter Decomposition in a Subtropical Mangrove

**DOI:** 10.3390/plants15030478

**Published:** 2026-02-03

**Authors:** Xinlei Xu, Yuxuan Wan, Zhiqiang Lu, Danyang Li, Li Ma

**Affiliations:** 1 Fisheries College, Jimei University, Xiamen 361021, China; 202421063037@jmu.edu.cn (X.X.); 202321061002@jmu.edu.cn (Y.W.); lidanyang@jmu.edu.cn (D.L.); 2 Third Institute of Oceanography, Ministry of Natural Resources, Xiamen 361005, China

**Keywords:** mangrove ecosystems, litter mixture, heavy metal accumulation, C/M ratio, ecological risk assessment

## Abstract

Mangrove ecosystems play a critical role in sequestering heavy metals pollutants, yet the dynamics of heavy metals accumulation during mixed litter decomposition remain poorly understood. This study investigated the seasonal and species-specific variations in heavy metals accumulation during the decomposition of *Kandelia obovata* (KO) and *Avicennia marina* (AM) leaf litter mixtures in a subtropical mangrove forest in the Jiulong River Estuary, Fujian, China. Using the litterbag technique, we monitored eight heavy metals (V, Cr, Ni, Cu, Zn, As, Se, Cd) across three mixing ratios (KO:AM = 1:2, 1:1, 2:1) in summer and winter. Results revealed that V concentrations were influenced by both season and litter ratio, with higher KO proportions enhancing V accumulation in summer but reducing it in winter. In contrast, Cr, Ni, Cu, As, Se, and Cd were primarily regulated by litter ratios: KO-dominated mixtures promoted Cr and Ni accumulation, while AM-dominated mixtures favored Cu, As, Se, and Cd. Zn exhibited the highest variability and was unaffected by season or ratio. Total organic carbon (TOC) and carbon/metal (C/M) ratios significantly correlated with reduced bioavailability of most heavy metals, whereas total nitrogen (TN) and C/N ratios showed no consistent relationship. The heavy metals accumulation index (*MAI*) indicated higher accumulation in summer than in winter, with the highest *MAI* observed in the KO:AM = 2:1 treatment group during summer (*MAI* = 1.36), whereas winter decomposition slowed accumulation rates. These findings highlight the dual regulatory roles of species composition and environmental factors in mangrove heavy metals cycling, offering critical insights for ecological risk assessment and contaminated soil remediation strategies in coastal ecosystems.

## 1. Introduction

With the rapid development of industrial and agricultural activities in modern river basins, coupled with population expansion and economic growth in coastal cities, estuarine environments are experiencing escalating degradation and pollution, posing direct threats to the survival and sustainability of regional ecosystems [1]. The combination of high pollutant tolerance and a capacity to buffer environmental stressors makes mangroves integral to estuarine ecosystems and has established mangrove pollution ecology as an emerging research focus [2]. Extensive studies by domestic and international scholars have investigated the environmental behaviors and ecological impacts of diverse pollutants—including heavy metals, petroleum hydrocarbons, and synthetic organic compounds—within mangrove ecosystems [3].

Mangrove wetlands are recognized as vital blue carbon ecosystems, playing a critical role in the global carbon cycle and contributing approximately 10% of the total organic carbon buried in coastal marine environments [4]. Furthermore, owing to their unique characteristics, mangrove wetlands efficiently accumulate heavy metals pollutants that are transported via tidal flows, river discharge, and stormwater runoff, surpassing the retention capacity of conventional tidal flats. Consequently, mangrove sediments frequently act as both sources and sinks for heavy metals contaminants [5]. Notably, interspecific differences exist in heavy metals uptake capacities among mangrove species. For instance, MacFarlane et al. demonstrated distinct distribution patterns of Cu, Zn, and Pb in *Avicennia marina*, with Zn and Pb predominantly localized in roots and minimally allocated to leaf litter [6]. While mangroves exhibit high tolerance to heavy metals, exceeding threshold concentrations can severely impair plant growth and morphological integrity.

Previous research on heavy metals pollution in mangrove ecosystems has primarily focused on four aspects: plant tolerance and heavy metals uptake mechanisms; sediment-based heavy metals enrichment and release dynamics; adsorption kinetics of heavy metals onto organic detritus; and phytoremediation potential of mangroves [7]. Although considerable research has focused on assessing contamination levels, ecological risks, source apportionment of heavy metals in mangrove sediments [8] and addressing heavy metals tolerance in plants and sediment-heavy metal interactions, limited attention has been paid to adsorption, enrichment, and release behaviors during mixed-litter decomposition of multi-species mangrove litter [9]. Given the natural coexistence of mangrove species in varying proportions and their interspecific interactions, elucidating how mixed-litter decomposition modulates heavy metal absorption, accumulation, and release is critical. Such insights are essential for understanding litter decomposition mechanisms and optimizing the environmental purification functions of mangrove ecosystems.

Therefore, this study aimed to elucidate how mixing ratios of *Kandelia obovata* and *A*. *marina* leaf litter and seasonal variations influence the partitioning, accumulation, and correlations of heavy metals (V, Cr, Ni, Cu, Zn, As, Se, Cd) during decomposition. We hypothesized that (1) the accumulation patterns of different heavy metals would respond distinctively to litter species composition and mixing ratios; (2) summer decomposition would promote higher heavy metal accumulation rates compared to winter due to enhanced microbial activity; and (3) key litter quality parameters (TOC, TN, C/M, C/N) would show significant correlations with heavy metal bioavailability, modulated by the litter mixture.

## 2. Materials and Methods

### 2.1. Study Area

**Figure 1:**
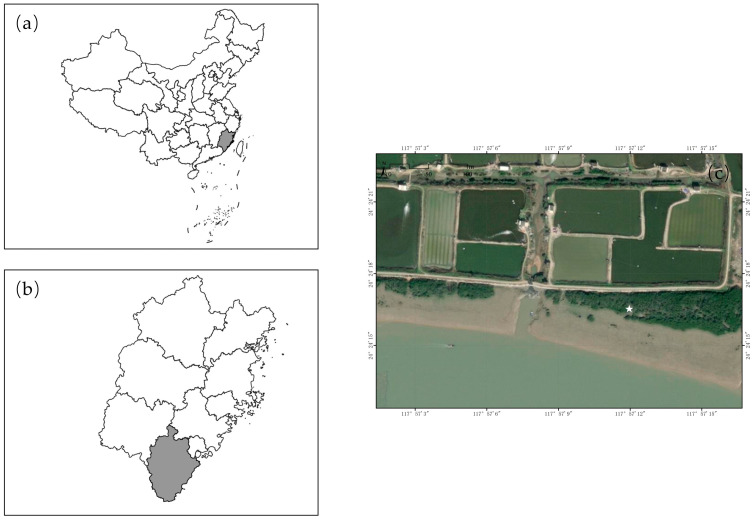
Location of the study area in the Mangrove Nature Reserve Jiulong River Estuary, Zhangzhou, Fujian. (**a**) The position of Fujian Province in China (in grey); (**b**) the position of Zhangzhou in Fujian Province (in grey); (**c**) Mangrove Nature Reserve of Jiulong River Estuary, Haimen Island, Fujian Province (white pentagram marks the location of the litterbags).

The study was conducted in the Mangrove Nature Reserve located at the Jiulong River Estuary, Zhangzhou City, Fujian Province, China (Figure 1). Characterized by a subtropical oceanic climate, the region experiences an annual mean temperature of 21 °C and receives 1400 mm of precipitation. Monthly temperatures range from 13 °C in January (coldest month) to 29 °C in July (warmest month), with relative humidity averaging 80% and annual sunshine duration approximating 2224 h. Semidiurnal tides dominate the area, exhibiting an average tidal range of 4 m. The mangrove vegetation is predominantly composed of *Kandelia obovata*, with scattered populations of *Avicennia marina*, *Aegiceras corniculatum* (Linn.) Blanco, *Bruguiera gymnorrhiza* (Linnaeus) Savigny, and other species.

Adjacent waters exhibit seasonal temperature fluctuations, ranging from 21.5–31.8 °C in summer to 16.1–19.0 °C in winter, with salinity levels varying between 12 and 26 [10,11]. Key water quality parameters include total phosphorus (0.09–0.12 mg/L), total nitrogen (1.72–2.36 mg/L), and chlorophyll *a* (0.74–8.67 μg/L) [11]. Phytoplankton biomass peaks in early spring, while cryptophytes and dinoflagellates dominate algal communities from September to December [9]. Sediments are primarily clayed silt, with soil organic carbon stocks in the upper 100 cm profile averaging 93.10 ± 11.28 kg C m^−2^ [12].

### 2.2. Experimental Setup

Newly senesced leaves of *K. obovata* and *A. marin* were harvested from the Jiulong River Estuary mangrove wetland on 10 July (summer) and 10 December (winter), respectively. The collected litter samples were air-dried for 48 h prior to experimental use. Decomposition dynamics were assessed using the litterbag method, wherein 36 g of dried litter was enclosed in 200 × 200 mm polyamide bags (1 mm mesh size). The experimental design comprised five treatments (Table 1).

Litterbags were deployed within a 20 × 20 m plot (24°24′16″ N, 117°57′12″ E) on mangrove surface sediment at the mid-tide level and secured to tree trunks using polyamide ropes. For each treatment, three replicate bags were randomly retrieved on days 7, 14, 21, 28, and 35 during summer, and days 7, 14, 21, 35, 49, and 77 during winter. Retrieved samples were gently rinsed with deionized water to remove adherent sediments, oven-dried at 80 °C to constant weight, and analyzed for total organic carbon (TOC) and heavy metal concentrations [13].

For heavy metal analysis, PTFE crucibles were sequentially cleaned with tap water, ultrapure water, and a 20% HNO_3_ solution under mild boiling for 5 h, followed by drying. Precisely 0.1000 g (±0.0001 g) of homogenized sample was transferred into a pre-treated crucible. After overnight equilibration with 3 mL HNO_3_ (GR grade), 5 drops of HF (AR grade) were added, and the mixture was gently agitated, sealed in a stainless-steel hydrothermal reactor, and digested at 190 °C for 24 h. Post-cooling, residual HF was evaporated on a hotplate. Subsequently, 2 mL HNO_3_ was added, and the sample was redigested at 150 °C for 8 h. The residue was dissolved in ultrapure water, transferred to a 50 mL volumetric flask, and diluted to volume. The final solution was filtered through a 0.45 μm membrane, and concentrations of V, Cr, Ni, Cu, Zn, As, Se, and Cd were quantified via inductively coupled plasma–mass spectrometry (ICP-MS, PerkinElmer, Waltham, MA, USA) [14]. The accuracy of the method was verified using certified reference materials (GBW10052). The recovery rates for all heavy metals were within the acceptable range (95–105 %), and the precision expressed as the relative standard deviation (RSD) of three replicates was generally below 10%.

### 2.3. Parameters’ Calculation

The comprehensive accumulation capacity of heavy metals in mixed *K. obovata* and *A. marina* litter was quantified using the Heavy Metal Comprehensive Accumulation Index (*MAI*) method [15], defined as

(1) $$MAI=[\frac{1}{N}]\times \sum_{j=1}^{N}I_{j}$$

where N denotes the number of heavy metal species analyzed (*N* = 8, corresponding to V, Cr, Ni, Cu, Zn, As, Se, and Cd), and $I_{j}=\bar{x}/\delta_{x}$*,* representing the ratio of the mean concentration ($\bar{x}$) of each heavy metal to its standard deviation (*δ_X_*).

### 2.4. Statistical Analysis

Statistical analyses were performed using Origin 2023b [16] for data visualization and SPSS 24.0 [17] for hypothesis testing. A three-way analysis of variance (ANOVA) was performed to investigate factor effects (season, treatment, and decomposition time) and interactions among them. When significant main effects were detected, post hoc pairwise comparisons were conducted using Tukey’s Honest Significant Difference (HSD) test. Normality of data distribution was verified via the Shapiro–Wilk test, and relationships between heavy metal concentrations, total organic carbon (TOC), total nitrogen (TN), carbon-to-metal (C/M), and carbon-to-nitrogen (C/N) ratios were evaluated using Pearson correlation analysis. Correlation coefficients (*r*) and significance levels (* *p* < 0.05, ** *p* < 0.01, *** *p* < 0.001; two-tailed) are reported in figures.

## 3. Results

### 3.1. Dynamic Changes in Heavy Metal Contents During Mixed-Litter Decomposition

Analysis of the three treatment groups revealed that, by the end of the summer decomposition period; the rank order of accumulated heavy metal contents for V, Cr, Zn, As, and Se remained consistent across all litter mixing ratios: Zn > V > As > Se > Cr. Notably, the magnitude of increase in Cu and Cd contents was positively correlated with the proportion of *A. marina* litter but inversely related to the proportion of *K. obovata* litter. Conversely, the accumulation of Ni increased with higher proportions of *K. obovata* litter and decreased with elevated *A. marina* litter content.

**Figure 2:**
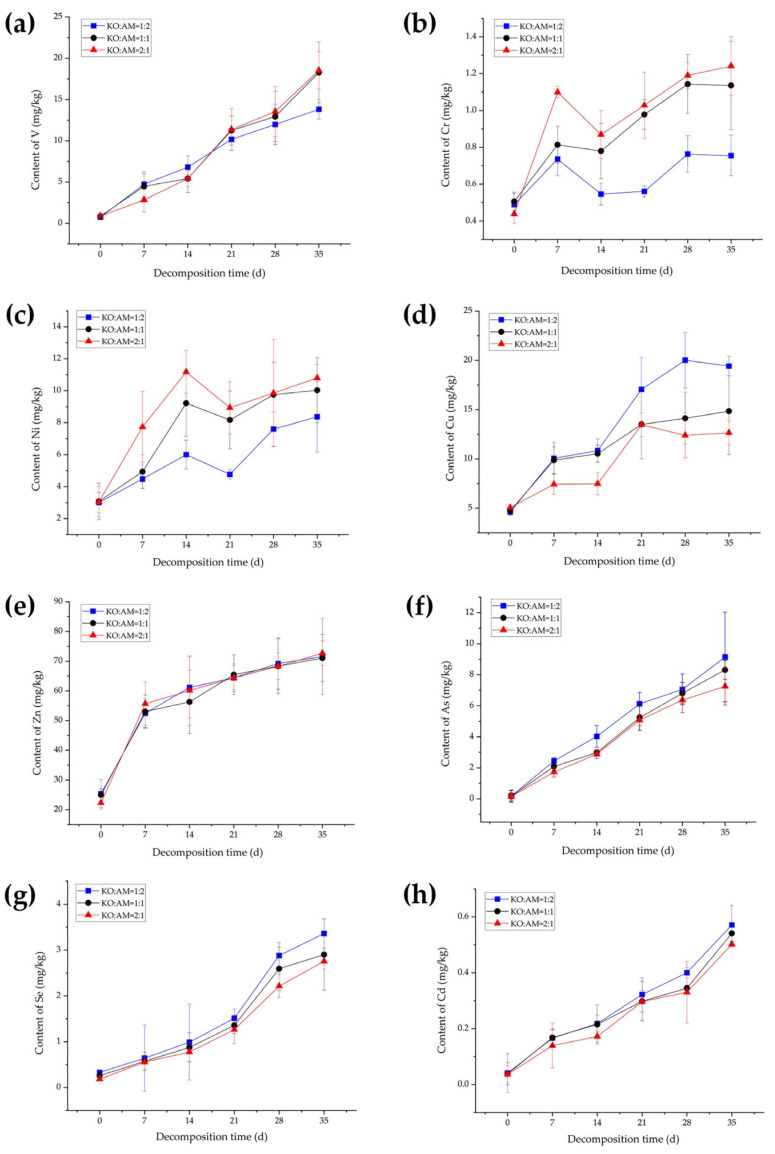
Contents of eight heavy metals, V (**a**), Cr (**b**), Ni (**c**), Cu (**d**), Zn (**e**), As (**f**), Se (**g**) and Cd (**h**), during the mixed-litter decomposition of *K. obovata* litter and *A. marina* litter in summer.

As revealed in Figure 2, with increasing decomposition time, the concentrations of most heavy metals exhibit a gradual upward trend, except for Cr, Ni, and Cu (Figure 2b–d), which displayed a fluctuating upward trajectory. Notably, V, Cr, and Ni (Figure 2a–c) showed distinct concentration differences across treatments after 35 days of summer decomposition, following the pattern KO:AM = 2:1 > 1:1 > 1:2. In contrast, Cu, As, Se, and Cd (Figure 2d,f–h) exhibited persistently higher concentrations in the KO:AM = 1:2 treatment throughout the decomposition period. By day 35, their concentrations ranked KO:AM = 1:2 > 1:1 > 2:1. Zn (Figure 2e) displayed no marked differences across treatments but demonstrated the highest variability. Final Zn concentrations increased by 46.19 mg/kg (KO:AM = 1:2), 46.10 mg/kg (KO:AM = 1:1), and 50.44 mg/kg (KO:AM = 2:1) relative to initial values, proportional influences on Zn accumulation were minimal compared to other heavy metals.

Analysis of winter decomposition data revealed accumulation patterns consistent with summer observations. The rank order of V, Cr, Zn, As, and Se contents remained unaffected by litter mixing ratios, while Cu, Cd, and Ni exhibited proportion-dependent trends. Specifically, higher proportions of *K. obovata* litter (KO:AM = 2:1) correlated with reduced accumulation of Cu and Cd but enhanced accumulation of Ni. Conversely, elevated *A. marina* litter content (KO:AM = 1:2) increased Cu and Cd accumulation while suppressing Ni accumulation.

**Figure 3:**
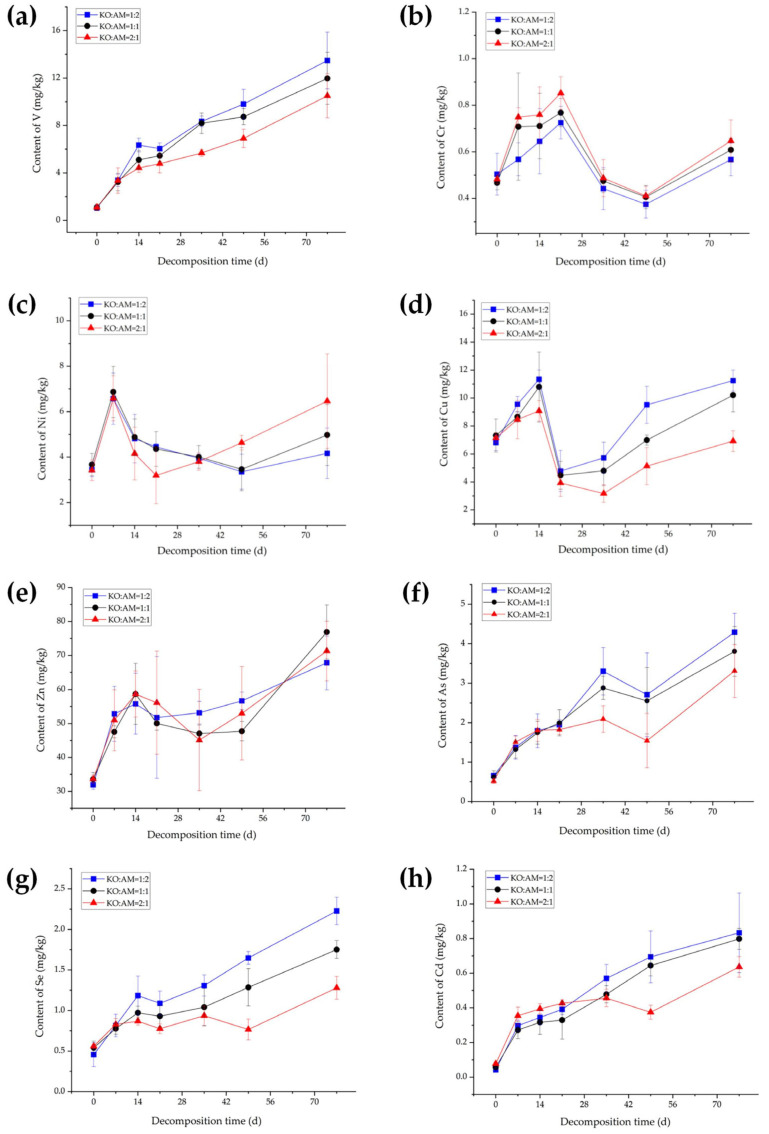
Contents of eight heavy metals, V (**a**), Cr (**b**), Ni (**c**), Cu (**d**), Zn (**e**), As (**f**), Se (**g**) and Cd (**h**), during the mixed-litter decomposition of *K. obovata* litter and *A. marina* litter in winter.

As shown in Figure 3, the amplitude of heavy metal concentration fluctuations during winter decomposition differs significantly from summer trends. Notably, V, Se, and Cd (Figure 3a,g,h) exhibit a gradual monotonic increase, whereas other heavy metals display multiphasic fluctuations (initial rise, decline, and subsequent rebound). Despite seasonal differences, the final concentrations of Cr and Ni (Figure 3b,c) after 77 days of winter decomposition followed the same hierarchy as in summer: KO:AM = 2:1 > 1:1 > 1:2. Similarly, Cu, As, Se, and Cd (Figure 3d,f–h) maintained the summer-derived order KO:AM = 1:2 > 1:1 > 2:1 by day 77. V (Figure 3a) exhibited divergent behavior compared to that of summer, with concentrations in the KO:AM = 1:2 treatment consistently exceeding those in other groups throughout winter decomposition. By day 77, V concentrations ranked KO:AM = 1:2 > 1:1 > 2:1, mirroring the trend observed for Cu, As, Se, and Cd. Zn (Figure 3e), while exhibiting the highest variability in winter, and differing from both summer patterns and other heavy metals, with final concentrations ordered KO:AM = 1:1 > 2:1 > 1:2. Net Zn accumulation increased by 43.31 mg/kg (KO:AM = 1:1), 37.70 mg/kg (KO:AM = 2:1), and 35.90 mg/kg (KO:AM = 1:2), highlighting its unique response to litter mixing ratios.

The observed trends in heavy metal accumulation were statistically validated by a three-way analysis of variance (Table 2). The results confirmed that season had a highly significant effect on the dynamics of V, As, Se, and Cd (*p* < 0.001), whereas it did not significantly influence Cr, Ni, Cu, or Zn. Litter mixture composition (treatment) significantly affected the concentrations of all metals except Zn (V: *p* < 0.05; others: *p* < 0.001). Decomposition time had a universally significant main effect on all eight metals (*p* < 0.001). Significant interactions were also detected, including S × T for Cu (*p* < 0.01) and T × D for V, Ni, Cu, and Cd (*p* < 0.05).

### 3.2. Correlation Among Heavy Metals, TN, TOC, Carbon/Metal (C/M) Ratios and Carbon/Nitrogen (C/N) Ratios

**Figure 4:**
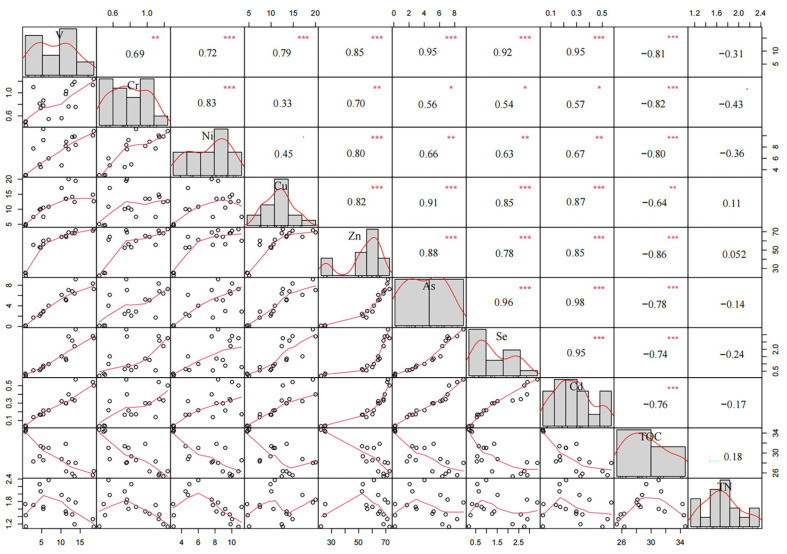
The Pearson’s correlation coefficients among several heavy metals during mixed-litter decomposition of litter in summer; *, ** and *** indicate correlation is significant at 0.05, 0.01 and 0.001 levels (2-tailed).

It can be seen from Figure 4 that TN in mixed litter exhibits no significant correlation with V, Cr, Ni, Cu, Zn, As, Se, Cd, or TOC during summer decomposition. In contrast, TOC demonstrates a highly significant negative correlation with all eight heavy metals (V, Cr, Ni, Cu, Zn, As, Se, Cd; *p* < 0.01), suggesting antagonistic interactions between TOC and heavy metal accumulation in summer litter mixtures. Notably, Cd shows strong positive correlations with V (*r* = 0.95), Cu (*r* = 0.87), Zn (*r* = 0.85), As (*r* = 0.98), and Se (*r* = 0.95) (*p* < 0.001), indicating synergistic co-accumulation of Cd with these heavy metals in summer. Cr exhibits no significant correlation with Cu (*r* = 0.33) but displays moderate correlations with As, Se, and Cd (*p* < 0.05), implying limited synergistic absorption among these elements. Ni shows significant positive correlations with V, Cr, Zn, As, Se, and Cd (*p* < 0.01), but no association with Cu (*r* = 0.45), highlighting its lack of synergistic interaction with Cu during summer decomposition.

**Figure 5:**
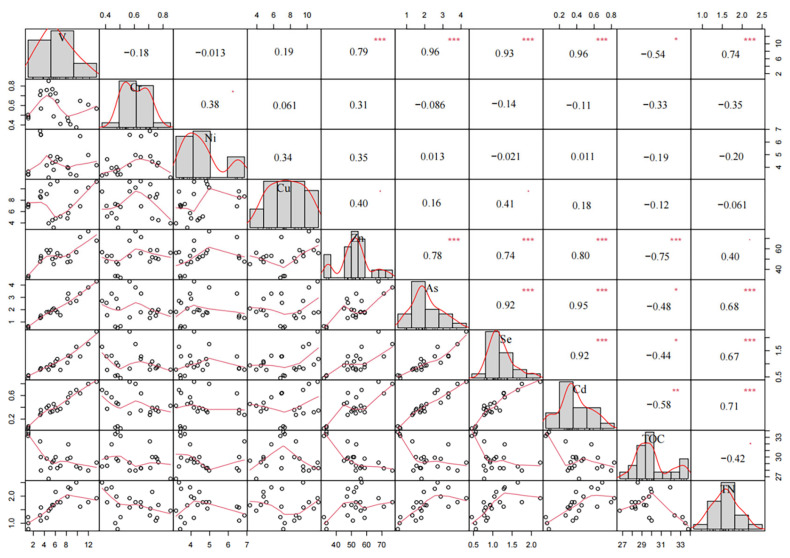
The Pearson’s correlation coefficients among several heavy metal during mixed-litter decomposition of litter in winter; *, ** and *** indicate correlation is significant at 0.05, 0.01 and 0.001 levels (2-tailed).

It can be seen from Figure 5 that TN exhibits significant positive correlations with V (*r* = 0.74), As (*r* = 0.68), Se (*r* = 0.67), and Cd (*r* = 0.71) during winter decomposition (*p* < 0.001), indicating synergistic co-accumulation of TN with these heavy metals. Conversely, TN shows no significant association with Cr, Ni, Cu, Zn, or TOC, suggesting minimal antagonistic interactions or independence in their absorption dynamics. TOC displays strong negative correlations with Zn (*r* = −0.75, *p* < 0.001) and Cd (*r* = −0.58, *p* < 0.01), signifying antagonistic effects on their accumulation. Weak negative correlations were observed between TOC and V, As, and Se (*p* < 0.05), while no significant relationships were detected with Cr, Ni, or Cu, further supporting limited antagonism between TOC and most heavy metals. Notably, Cr, Ni, and Cu showed no significant correlations with other heavy metals, whereas V, Zn, As, Se, and Cd demonstrated strong mutual positive correlations (*p* < 0.001). This highlights the synergistic co-accumulation of the latter group in winter litter mixtures, contrasting with the independence of Cr, Ni, and Cu in absorption dynamics.

Table 3 summarizes the correlation analysis between eight heavy metal concentrations (V, Cr, Ni, Cu, Zn, As, Se, and Cd) and carbon-to-metal (C/M) or carbon-to-nitrogen (C/N) ratios during the decomposition of *K. obovata* litter and *A. marina* leaf litter across different mixing ratios.

In the KO:AM = 1:2 treatment group, all heavy metals except Cr demonstrated significant negative correlations with the C/M ratio (Table 3). Specifically, Ni, Cu, and Cd exhibited moderate correlations (coefficients: −0.614, −0.681, and −0.645, respectively; *p* < 0.05), while V, Zn, As, and Se showed stronger correlations (coefficients: −0.804, −0.963, −0.705, and −0.690, respectively; *p* < 0.01). These results suggest that elevated C/M ratios under the KO:AM = 1:2 ratio correlate with reduced heavy metal concentrations, excluding Cr. For the KO:AM = 1:1 group, all heavy metals displayed significant negative correlations with C/M: Cr, Ni, and Cd exhibited coefficients of −0.620, −0.631, and −0.635 (*p* < 0.05), whereas V, Cu, Zn, As, and Se showed stronger associations (−0.772, −0.694, −0.930, −0.746, and −0.724; *p* < 0.01), indicating a consistent inverse relationship between C/M and heavy metal concentrations. In the KO:AM = 2:1 group, all heavy metals except Cu negatively correlated with C/M, with Cr, Ni, Se, and Cd showing moderate coefficients (−0.667, −0.647, −0.646, and −0.634; *p* < 0.05) and V, Zn, and As exhibiting stronger values (−0.686, −0.953, and −0.704; *p* < 0.01).

For C/N ratios, significant negative correlations were limited to specific heavy metals depending on the mixing ratio. Under KO:AM = 1:2, V, Zn, and Cd concentrations decreased with increasing C/N (coefficients: −0.605, −0.666, and −0.604; *p* < 0.05), while Cr, Ni, Cu, As, and Se showed no significant relationship. In the KO:AM = 1:1 group, only Cd exhibited a negative correlation with C/N (coefficient: −0.603; *p* < 0.05). Conversely, under KO:AM = 2:1, Zn was the sole heavy metal inversely linked to C/N (coefficient: −0.608; *p* < 0.05), with no significant associations observed for other heavy metals.

### 3.3. Dynamic Changes in the Heavy Metals Comprehensive Accumulation Index (MAI) During Mixed-Litter Decomposition

**Figure 6:**
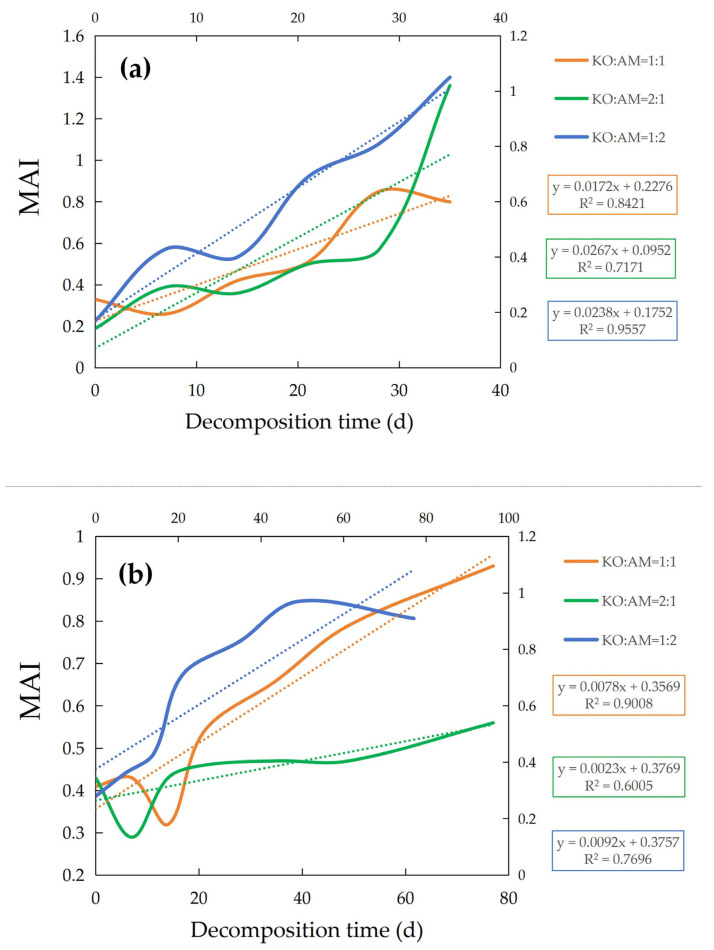
Variations in V, Cr, Ni, Cu, Zn, As, Se and Cd. The heavy metals’ comprehensive accumulation index (*MAI*) during mixed-litter decomposition of *K. obovata* litter and *A. marina* litter in summer (**a**) and winter (**b**).

It is depicted in Figure 6 that the temporal dynamics of the Heavy Metal Comprehensive Accumulation Index (*MAI*) for *K. obovata and A. marina* leaf litter decomposed under different mixing ratios during summer and winter. Each figure comprises two components: the *MAI* trajectory (solid line) and its corresponding trend line (dotted line). Seasonal *MAI* trends are categorized into three mixing ratios, denoted by distinct colors: KO:AM = 1:2 (blue), KO:AM = 1:1 (orange), and KO:AM = 2:1 (green).

As shown in Figure 6a, *MAI* values in all summer treatment groups exhibited a time-dependent upward trend, albeit with varying accumulation rates. The KO:AM = 1:1 group displayed the slowest increase (slope = 0.0172), followed by KO:AM = 1:2 (slope = 0.0238) and KO:AM = 2:1 (slope = 0.0267), indicating differential accumulation kinetics across ratios. Specifically, the *MAI* for KO:AM = 1:2 rose from 0.17 (day 0) to 1.05 (day 35), reflecting a pronounced accumulation over time. Similarly, KO:AM = 1:1 showed a gradual increase from 0.33 to 0.80 during the same period, while KO:AM = 2:1 demonstrated the highest final *MAI* (1.36 at day 35), rising from an initial value of 0.19. These results suggest that a higher proportion of *K. obovata* litter (KO:AM = 2:1) may enhance heavy metal accumulation during summer decomposition.

In winter (Figure 6b), *MAI* trends diverged markedly from summer patterns. Accumulation slopes were substantially lower across all groups, ordered as KO:AM = 1:2 (0.0092) > KO:AM = 1:1 (0.0078) > KO:AM = 2:1 (0.0023). For KO:AM = 1:2, the *MAI* increased modestly from 0.28 (day 0) to 0.91 (day 77), reflecting slower kinetics compared to summer. Similarly, KO:AM = 1:1 showed a rise from 0.41 to 0.93, with a higher initial value but comparable final accumulation. In contrast, KO:AM = 2:1 exhibited minimal growth (0.43 to 0.56), yielding the lowest winter *MAI* among all groups. This stark seasonal contrast—where KO:AM = 2:1 displayed the highest summer *MAI* but the lowest winter *MAI*—highlights the modulating role of environmental conditions on litter-mediated heavy metal accumulation.

## 4. Discussion

### 4.1. Dynamic Analysis of Heavy Metal Contents

Mangrove plants play a dual role in heavy metal dynamics, serving both as suppliers of essential trace elements for growth and mitigators of environmental contamination through heavy metal adsorption [18,19]. This study elucidates the biogeochemical cycling of heavy metals during *K. obovata* and *A. marina* leaf litter decomposition in subtropical mangrove ecosystems. The three-way ANOVA (Table 2) delineated the key statistical drivers of metal dynamics. Season was a dominant factor for V, As, Se, and Cd (*p* < 0.001), underscoring the role of temperature in enhancing their summer accumulation. In contrast, litter composition (Treatment) exerted a strong, significant effect on the accumulation of Cr, Ni, Cu, As, Se, and Cd (*p* < 0.001), confirming our first hypothesis regarding species-specific controls. Notably, the significant S × T interaction for Cu (*p* < 0.01) revealed that the effect of litter mixing on Cu was contingent on seasonal conditions. Furthermore, the significant T × D interactions for V, Ni, Cu, and Cd (*p* < 0.05) indicate that the influence of litter composition evolved throughout decomposition. The exception was Zn, which was unaffected by either season or treatment, highlighting its distinct biogeochemical behavior. These statistical results collectively affirm that metal accumulation is differentially regulated by an interplay of environmental forcing, litter quality, and temporal dynamics. Seasonal disparities in heavy metal dynamics—notably higher accumulation rates in summer than winter—emphasize temperature-driven modulation of decomposition rates and heavy metal mobility [20,21]. Furthermore, heavy metal-specific behaviors reflect complex interactions between biotic (e.g., microbial activity) and abiotic drivers (e.g., sediment chemistry, such as pH and EC) [22,23]. For instance, V, Cr, Zn, As, and Se concentrations showed no dependence on litter mixing ratios, whereas Cu, Cd, and Ni accumulation correlated strongly with KO:AM proportions (Figure 6). This divergence likely stems from differential heavy metal adsorption capacities in sediments and their affinity for organic matter [24,25]. Additionally, microplastics prevalent in coastal environments may act as vectors for heavy metals, adsorbing and transporting them into the litter–sediment matrix, potentially altering accumulation patterns across treatments [4]. Oscillatory trends in Cr, Ni, and Cu concentrations (e.g., initial rise followed by decline and subsequent rebound) may reflect dynamic equilibria between heavy metal release during decomposition and re-adsorption/precipitation processes mediated by redox fluctuations or ligand interactions [26]. Such fluctuations could also be influenced by microbial community shifts induced by heavy metal stress, which in turn affect decomposition pathways and metal speciation [27].

Notably, heavy metal accumulation exhibited distinct dependencies: V levels responded to both season and litter ratio; Cr, Ni, Cu, As, Se, and Cd were ratio-dependent; and Zn remained unaffected by either factor (Table 3). Such variability aligns with the unique physicochemical properties of each heavy metal, which govern their environmental behavior and bioavailability [28,29]. For example, Zn’s high mobility and role as a microbial micronutrient [30,31] may explain its pronounced variability despite nonsignificant intergroup differences. Early stage increases in most heavy metals (excluding V, Se, Cd) likely correspond to organic matter degradation liberating bound heavy metals [32]. Subsequent fluctuations suggest geochemical transformations, such as shifts in pH or redox conditions favoring re-adsorption or precipitation into less bioavailable forms [33]. In contrast, the gradual accumulation of V, Se, and Cd implies slow release from recalcitrant organic complexes within litter matrices [34].

Critically, KO-dominated ratios (e.g., KO:AM = 2:1) enhanced heavy metal release in summer but suppressed it in winter, while AM-rich mixtures exhibited opposing effects. These findings underscore species-specific litter chemistry as a determinant of heavy metal dynamics—consistent with prior reports that mangrove species differentially modulate heavy metal binding and release due to variations in biochemical composition (e.g., lignin content, tannin profiles) [35]. This highlights the ecological significance of mangrove species composition in regulating heavy metal cycling and mitigating contamination risks.

### 4.2. Correlation Analysis of Heavy Metals

The correlation analysis in this study reveals significant relationships among heavy metals (V, Cr, Ni, Cu, Zn, As, Se, and Cd) and between heavy metals and soil properties (TN, TOC, C/M, and C/N ratios); these findings enhance understanding of the mechanisms governing heavy metal dynamics during leaf litter decomposition. During summer mixed-litter decomposition, no significant correlations were observed between Cu absorption and Cr or Ni absorption. In contrast, all other heavy metals (V, Zn, As, Se, and Cd) exhibited positive correlations, suggesting synergistic interactions during absorption and accumulation. This phenomenon may arise from similarities in their chemical properties and geochemical behaviors, potentially leading to co-adsorption or co-precipitation [36,37]. Such synergies have critical implications for pollution assessments, as the presence of one heavy metal may signal co-occurrence of others. Synergistic interactions also occur when the combined effect of two or more heavy metals is greater than the sum of their individual effects [23]. This can arise from mutual enhancement of toxicity or shared biological pathways [38,39]. Microorganisms can also play a role in synergistic biodetoxification, where the presence of certain co-existing pollutants, such as Se, can lower the ecotoxicity of heavy metals like Cu, a process potentially activated by substances like anthraquinone-2,6-disulfonate (AQDS) through alterations in the coordination environment of outer-membrane proteins [40]. During winter decomposition, Cu, Cr, and Ni showed no significant correlations with V, Zn, As, Se, or Cd, indicating that heavy metal interactions are context-dependent and influenced by chemical affinities and seasonal environmental conditions [41]. Seasonal variations in heavy metal absorption capacities may weaken or reverse correlations between heavy metals in litter [41]. Overall, heavy metal interactions can shift between competitive and synergistic modes, contingent on elemental specificity and environmental context [42]. Notably, summer exhibited more pronounced heavy metal absorption during mixed-litter decomposition, likely due to monsoon rains, typhoon activity, and intensified intertidal fauna activity in the study area [43].

No significant correlations were detected between TN and heavy metals during summer decomposition of mixed *K. obovata* and *A. marina* litter in the Jiulong River Estuary. In winter, however, TN absorption displayed synergistic effects with V, As, Se, and Cd. TOC exhibited negative correlations with heavy metals during summer decomposition, implying competitive sorption mechanisms where organic matter preferentially binds heavy metals, reducing their bioavailability [44]. This aligns with prior studies highlighting organic matter’s role in immobilizing soil heavy metals [45]. In winter, TOC absorption showed antagonistic effects only toward Zn and Cd, with weak or no correlations to other heavy metals, consistent with observations at the *K. obovata* site where TN and TOC correlations with heavy metals varied (positive, negative, or absent) [19].

A significant negative correlation emerged between the C/M ratio and heavy metal concentrations (Table 3), suggesting that higher C/M ratios correspond to reduced heavy metal bioavailability and mobility [41]. Elevated C/M ratios reflect complex organic matter compositions that enhance heavy metal binding, limiting uptake by biota [46]. These results corroborate earlier findings linking increased organic matter content to decreased heavy metal bioavailability [47]. Thus, the C/M ratio may serve as a critical regulatory parameter for soil heavy metal concentrations, implying that adjusting this ratio via organic amendments could mitigate heavy metal toxicity in contaminated soils [48]. In contrast, the C/N ratio showed no significant correlation with most heavy metals. The mixing ratio of *K. obovata* and *A. marina* litter significantly influenced these relationships: (*i*) KO:AM = 1:1: C/M ratio negatively correlated with all heavy metals; C/N ratio negatively correlated only with with Cd; (*ii*) KO:AM = 1:2: Cr concentration was unaffected by C/M, while V, Zn, and Cd correlated with C/N; (*iii*) KO:AM = 2:1: Cu concentration was independent of C/M, whereas Zn and Cd correlated with C/N. This variability likely stems from differences in organic matter composition and heavy metal-binding properties between the two litter types [49]. These findings hold ecological significance for ecosystems impacted by heavy metal pollution, as litter composition may regulate heavy metal bioavailability, informing restoration strategies; however, the mechanisms underlying C/M ratio effects on heavy metal concentrations require further exploration.

### 4.3. Change in Comprehensive Accumulation Index of Heavy Metals

The comprehensive accumulation index (*MAI*) serves as a valuable tool for evaluating the integrated accumulation capacity of heavy metals in mangrove ecosystems. The observed temporal increase in *MAI* during this study highlights progressive heavy metal accumulation in decomposing leaf litter, underscoring the critical role of mangrove ecosystems in heavy metal sequestration [50]. Seasonal comparisons revealed distinct *MAI* patterns: during summer, *MAI* values for all litter mixing ratios increased steadily, peaking at day 35 (Figure 6). This trend suggests enhanced heavy metal accumulation during summer decomposition, likely driven by elevated temperatures and microbial activity that promote heavy metal release from decomposing litter [50]. In contrast, winter decomposition exhibited a muted *MAI* increase, plateauing by day 35, potentially due to reduced microbial activity and slower decomposition rates under colder conditions [51]. These seasonal disparities emphasize the necessity of accounting for environmental variables when evaluating ecological risks linked to heavy metal accumulation in decomposing litter.

Variations in *MAI* across different mixing ratios of *K. obovata* and *A. marina* litter further highlight the influence of species interactions on heavy metal accumulation. Non-uniform *MAI* values across mixing proportions indicate that litter composition critically governs accumulation dynamics. For example, lower *MAI* values in treatments dominated by *A. marina* litter may reflect its distinct decomposition pathways and greater capacity to immobilize heavy metals [52]. Conversely, the *K. obovata* dominant mixture (KO:AM = 2:1) exhibited the highest summer *MAI*, suggesting species-specific contributions to heavy metal accumulation, potentially mediated by differences in litter quality (e.g., lignin, cellulose, and phenolic compound content) [53,54]. These findings imply that adjusting plant species ratios in decomposition studies or natural ecosystems could modulate heavy metal release and mitigate environmental impacts [55]. Strategically selecting species with higher accumulation capacities may enhance the natural filtration efficiency of mangrove ecosystems.

## 5. Conclusions

This study investigated the dynamics of heavy metal accumulation during mixed leaf litter decomposition in a subtropical mangrove forest ecosystem. The results showed that (*i*) the temporal patterns of metal accumulation differed significantly between seasons. V accumulation was uniquely sensitive to both season and mixing ratio, with KO-dominated mixtures (2:1) enhancing accumulation in summer but reducing it in winter. In contrast, the accumulation of Cr and Ni was consistently promoted by higher proportions of KO litter, whereas Cu, As, Se, and Cd were preferentially retained in AM-dominated mixtures (1:2), regardless of season. Zinc exhibited the highest net increase (e.g., up to 50.44 mg/kg in summer); (*ii*) with the exception of Cu, Cr, and Ni, all other heavy metals (V, Zn, As, Se, and Cd) displayed significant positive correlations. Significant negative correlations were observed between TOC, the C/M ratio, and most heavy metal concentrations (e.g., Zn vs. C/M, *r* up to −0.963); TN in summer and the C/N ratio showed no significant associations with heavy metal concentrations; (*iii*) the Heavy Metal Comprehensive Accumulation Index (*MAI*) quantitatively confirmed that accumulation was significantly greater in summer (peak *MAI* = 1.36 for KO:AM = 2:1) than in winter, aligning with faster decomposition rates. The highest *MAI* in the KO-dominated treatment during summer indicated that *K. obovata* litter, under favorable conditions, drove the net accumulation of the studied heavy metal suite. To further improve the ecological relevance of these findings, future studies should incorporate phytoaccessibility assays to distinguish between total and bioavailable metal pools, thereby enhancing ecological risk assessments. The potential influences of micro-environment factors on decomposition of different litters should also be further investigated.

## Figures and Tables

**Table 1:** Two single species and three mixed species treatments in the litter decomposition experiment.

Treatment Group		Abbreviation	Weight of Samples (g)	
Single species treatments	*Kandelia obovata*	KO	KO = 36	
	*Avicennia marina*	AM	AM = 36	
Mixed species treatments	*Kandelia obovata*:*Avicennia marina* = 1:2	KO:AM = 1:2	KO = 12	AM = 24
	*Kandelia obovata*:*Avicennia marina* = 1:1	KO:AM = 1:1	KO = 18	AM = 18
	*Kandelia obovata*:*Avicennia marina* = 2:1	KO:AM = 2:1	KO = 24	AM = 12

**Table 2:** Results of three-way factorial ANOVA (F-values and significance) for the effects of Season (S), Treatment (T), Decomposition time (D) and their interactions on concentrations of heavy metals during litter decomposition.

Heavy Metal	Season (S)	Treatment (T)	Decomposition Time (D)	S × T	S × D	T × D
V	F = 54.91 *p* < 0.001	F = 4.33 *p* < 0.05	F = 11.30 *p* < 0.001	F = 2.59 *p* = n.s.	F = 5.21 *p* < 0.001	F = 2.04 *p* < 0.05
Cr	F = 1.73 *p* = n.s.	F = 16.11 *p* < 0.001	F = 7.78 *p* < 0.001	F = 1.60 *p* = n.s.	F = 8.57 *p* < 0.001	F = 1.11 *p* = n.s.
Ni	F = 0.02 *p* = n.s.	F = 14.97 *p* < 0.001	F = 5.48 *p* < 0.001	F = 0.91 *p* = n.s.	F = 5.69 *p* < 0.001	F = 2.31 *p* < 0.05
Cu	F = 2.72 *p* = n.s.	F = 20.94 *p* < 0.001	F = 38.10 *p* < 0.001	F = 6.85 *p* < 0.01	F = 14.82 *p* < 0.001	F = 6.15 *p* < 0.001
Zn	F = 0.76 *p* = n.s.	F = 3.04 *p* = n.s.	F = 19.10 *p* < 0.001	F = 0.29 *p* = n.s.	F = 9.67 *p* < 0.001	F = 0.55 *p* = n.s.
As	F = 31.33 *p* < 0.001	F = 10.42 *p* < 0.001	F = 44.70 *p* < 0.001	F = 1.50 *p* = n.s.	F = 22.43 *p* < 0.001	F = 2.11 *p* = n.s.
Se	F = 35.62 *p* < 0.001	F = 12.74 *p* < 0.001	F = 47.76 *p* < 0.001	F = 0.21 *p* = n.s.	F = 19.70 *p* < 0.001	F = 2.07 *p* = n.s.
Cd	F = 22.21 *p* < 0.001	F = 12.96 *p* < 0.001	F = 39.61 *p* < 0.001	F = 0.34 *p* = n.s.	F = 10.26 *p* < 0.001	F = 3.88 *p* < 0.05

**Table 3:** Correlation coefficients between heavy metal concentrations and C/M ratios and C/N ratios.

Group		V	Cr	Ni	Cu	Zn	As	Se	Cd
KO:AM = 1:2	C/M	−0.804 **	−0.394	−0.614 *	−0.681 *	−0.963 **	−0.705 **	−0.690 **	−0.645 *
	C/N	−0.605 *	−0.087	−0.197	−0.24	−0.666 *	−0.399	−0.381	−0.604 *
KO:AM = 1:1	C/M	−0.772 **	−0.620 *	−0.631 *	−0.694 **	−0.930 **	−0.746 **	−0.724 **	−0.635 *
	C/N	−0.449	−0.137	−0.034	−0.088	−0.483	−0.319	−0.296	−0.603 *
KO:AM = 2:1	C/M	−0.686 **	−0.667 *	−0.647 *	−0.508	−0.953 **	−0.704 **	−0.646 *	−0.634 *
	C/N	−0.378	−0.327	−0.367	0.021	−0.608 *	−0.345	−0.200	−0.485

* and ** indicate correlation is significant at 0.05 and 0.01 levels (2-tailed).

## Data Availability

Data are contained within the article.
